# Nutritional Assessment and Management of Patients with Brain Neoplasms Undergoing Neurosurgery: A Systematic Review

**DOI:** 10.3390/cancers17050764

**Published:** 2025-02-24

**Authors:** Jose Carlos Pebes Vega, Stefano Mancin, Giulia Vinciguerra, Elena Azzolini, Francesco Colotta, Manuela Pastore, Sara Morales Palomares, Diego Lopane, Giovanni Cangelosi, Simone Cosmai, Daniela Cattani, Riccardo Caccialanza, Emanuele Cereda, Beatrice Mazzoleni

**Affiliations:** 1 Department of Biomedical Sciences, Humanitas University, Via Rita Levi Montalcini 4, 20090 Pieve Emanuele, Milan, Italy; josecarlos.pebesvega@st.hunimed.eu (J.C.P.V.); stefano.mancin@humanitas.it (S.M.); elena.azzolini@humanitas.it (E.A.); francesco.colotta@humanitasresearch.it (F.C.); diego.lopane@hunimed.eu (D.L.); simone.cosmai@hunimed.eu (S.C.); daniela.cattani@humanitas.it (D.C.); beatrice.mazzoleni@hunimed.eu (B.M.); 2 IRCCS Humanitas Research Hospital, Via Manzoni 56, 20089 Rozzano, Milan, Italy; giulia.vingiguerra@humanitas.it (G.V.); manuela.pastore@humanitas.it (M.P.); 3 Department of Pharmacy, Health and Nutritional Sciences (DFSSN), University of Calabria, 87036 Rende, Italy; sara.morales@unical.it; 4 Unit of Diabetology, Asur Marche—Area Vasta 4 Fermo, 63900 Fermo, Italy; giovanni.cangelosi@virgilio.it; 5 Clinical Nutrition and Dietetics Unit, Fondazione IRCCS Policlinico San Matteo, 27100 Pavia, Italy; e.cereda@smatteo.pv.it

**Keywords:** brain neoplasms, nutritional status, pre-habilitation, systematic review

## Abstract

Background/Aim: Nutritional management in neurosurgical patients with brain neoplasms is critical, as optimal nutritional status is potentially associated with improved clinical outcomes. This systematic review aimed to analyze the impact of pre- and postoperative nutritional assessment and effect of prepost interventions on the clinical outcomes. Methods: A systematic review was conducted using the PubMed, Cochrane Library, Embase, and CINAHL databases, complemented by a search of grey literature. Study quality was assessed using the Joanna Briggs Institute framework, and the certainty of evidence was graded according to the Oxford Centre for Evidence-Based Medicine levels. Results: Fourteen studies, encompassing a total of 11,224 adult patients with brain neoplasms, were included. Many of these studies were retrospective, had small sample sizes, and examined diverse nutritional protocols. Preoperative nutritional status assessment, including clinical parameters such as albumin (*p* < 0.001), Controlling Nutritional Status score (*p* = 0.001), and Prognostic Nutritional Index (*p* < 0.010), combined with postoperative oral nutritional supplements (*p* < 0.001), was significantly associated with postoperative clinical outcomes. Additionally, personalized nutritional counseling contributed to a reduction in complications and facilitated more effective functional recovery. Conclusions: Nutritional care is vital in managing neurosurgical patients with brain neoplasms, reducing complications and enhancing postoperative recovery and overall clinical outcomes. A multidisciplinary team is key to optimal outcomes. Future research should aim to standardize protocols for broader applicability.

## Simple Summary

Brain tumors are the second most common cancer among adolescents and young adults and the eighth most common cancer among older adults. Symptoms vary depending on the size and location of the tumor and often include headaches, seizures, and motor, visual, and neurological deficits, all of which significantly impact daily life activities. The standard treatment for brain tumors involves a multifocal approach, combining surgery, radiation therapy, and chemotherapy. Research has identified nutritional status as a critical determinant of surgical outcomes. Malnourished patients are more likely to experience postoperative complications, such as infections, delayed healing, and extended hospital stays. Therefore, a proper pre- and postoperative nutritional strategy is essential for the effective clinical management of these patients.

## 1. Introduction

Brain neoplasms are abnormal tissue masses characterized by uncontrolled cellular growth [1,2], which can originate in any part of the central nervous system (CNS) or peripheral nervous system (PNS) [3,4]. These neoplasms are classified into primary tumors, arising directly in the CNS [5], and secondary tumors, which metastasize from malignancies in other organs, such as the lungs, breast, or skin [6]. They can also be categorized as benign tumors, which are slow-growing and non-invasive [7,8], or malignant tumors [3], characterized by aggressive and infiltrative behavior. From an epidemiological perspective, brain neoplasms are the second most common cancer among adolescents and the eighth most frequent among older adults [9,10]. Globally, the incidence of brain cancer is approximately 4.8 cases per 100,000 in men and 3.6 per 100,000 in women. In Europe, age-standardized rates (ASR) are among the highest, with 7.9 cases per 100,000 in men and 5.6 per 100,000 in women [11].

The clinical impact of brain neoplasms on survival and quality of life is profound. This is largely determined by their biological aggressiveness and location [12], which influence symptoms and therapeutic strategies. Common symptoms include headaches, seizures, neurological deficits, and cognitive impairments, often linked to increased intracranial pressure [13]. These clinical challenges necessitate a multidisciplinary approach to management. For example, glioblastoma multiforme (GBM), the most aggressive form of brain tumor [13,14], poses significant therapeutic challenges due to its invasiveness, high mortality rate, and recurrence propensity. Standard treatment for brain tumors involves a multimodal approach combining surgery [15,16,17], radiotherapy [18,19], and pharmacotherapy [20].

Nutritional assessment and management play a crucial role in neurosurgical care [21], particularly for patients with brain neoplasms. Malnutrition [22], a frequently overlooked condition, has a significant impact on clinical outcomes, increasing the risk of postoperative complications, infections, delayed wound healing, and prolonged hospital stays [23,24]. Its prevalence among cancer patients ranges between 20% and 70% [25], driven by inadequate caloric intake, metabolic alterations, and systemic inflammation induced by the tumor and its treatments [26]. In patients with brain tumors, symptoms such as nausea, vomiting, and dysphagia exacerbate nutritional deficiencies, heightening the risk of complications. Addressing these issues requires timely and comprehensive nutritional assessment to identify at-risk patients and implement targeted interventions [27]. Validated screening tools, including the Subjective Global Assessment (SGA) [28], Controlling Nutritional Status (CONUT) [29], and Prognostic Nutritional Index (PNI) [30], along with biochemical parameters (e.g., albumin, lymphocytes, and cholesterol), anthropometric measures, and medical history, enable a holistic evaluation of nutritional status. These tools also facilitate the monitoring of therapeutic interventions over time [31]. In neurosurgical practice, nutritional support is integral to care protocols such as the Enhanced Recovery After Surgery (ERAS) program [32], which emphasizes pre- and postoperative nutritional management. Prehabilitation programs, incorporating personalized nutritional interventions to optimize preoperative status, have demonstrated significant improvements in clinical outcomes [33]. The use of oral nutritional supplements (ONS) [34], including maltodextrins [35] and immunonutrients such as glutamine, arginine, and omega-3 fatty acids, has been shown to reduce inflammation, modulate immunity, and enhance recovery [36]. A key aspect of effective nutritional management is the incorporation of rapid and accurate screening tools into routine clinical practice. Nutritional assessments should be conducted within the first 24–48 h of hospitalization and repeated periodically throughout the postoperative period [37]. Using validated tools and biochemical markers ensures the continuous optimization of perioperative care. Furthermore, a multidisciplinary team involving neurosurgeons, nutritionists, and nurses is essential for delivering comprehensive and personalized care [38]. Given the evidence, targeted nutritional support is a cornerstone of care for neurosurgical patients with brain neoplasms. Accurate assessment and timely intervention not only reduce postoperative complications but also enhance quality of life and survival outcomes.

### Objectives of the Review

This systematic review (SR) aims to evaluate nutritional assessment and treatment strategies in patients undergoing neurosurgical procedures for brain neoplasms. Secondary objectives include identifying the healthcare professionals involved in nutritional management and analyzing follow-up protocols to improve clinical outcomes.

## 2. Materials and Methods

### 2.1. Review Methodology

This SR was reported in accordance with the Preferred Reporting Items for Systematic Reviews and Meta-Analyses (PRISMA) guidelines [39].

### 2.2. Registration of the Systematic Review Protocol

The protocol for this SR was registered in the International Prospective Register of Systematic Reviews (PROSPERO) under the National Institute of Health Research, accessible at https://www.crd.york.ac.uk/prospero/ (accessed on 19 February 2025) with the registration number CRD42024572727.

### 2.3. Research Question Formulation

The research question addressed was as follows: What is the efficacy of pre- and postoperative nutritional assessment and interventions in improving clinical and functional outcomes in patients with brain neoplasms undergoing neurosurgical procedures?

This question was structured using the PICO framework [40], which emphasizes four key components: Population (P), Problem (p), Intervention (I), Comparison (C), and Outcome (O). P: Adults with brain neoplasms undergoing cranial neurosurgery; I: Nutritional assessment and interventions; C: Standard care or no nutritional assessment interventions; O: Identification of healthcare professionals involved, and postoperative outcomes, including recovery time, quality of life, and complication rates.

### 2.4. Search Strategy

A comprehensive and systematic literature search was conducted between August and September 2024 to identify contemporary sources on nutritional assessment interventions in neurosurgical oncology patients. The bibliographic search was conducted using the PubMed, Cochrane Library, CINAHL, and Embase databases, supplemented by a search of grey literature sources, including Google Scholar. The initial search was conducted in individual databases without time restrictions between May and June 2024 by two researchers (JCPV and SM). Search terms were derived through an initial analysis of the MeSH thesaurus of the National Library of Medicine and its corresponding synonyms. These included keywords such as “brain neoplasms”, “brain tumors”, “nutritional status”, and “prehabilitation”, along with relevant synonyms and related phrases. Boolean operators (AND, OR) were used to ensure a broad yet targeted search (Supplementary File S1). Titles and abstracts from the initial screening were independently evaluated using EndNote 21 by two researchers (JCPV and GV). In case of disagreement, a third researcher was consulted to resolve discrepancies (SM). EndNote 21 (https://endnote.com/) was also used to systematically remove duplicates and irrelevant records. Full-text articles for potentially relevant studies were retrieved and rigorously reviewed for eligibility according to predefined inclusion and exclusion criteria.

### 2.5. Inclusion and Exclusion Criteria

Inclusion criteria comprised primary studies published in English, studies involving adult patients (≥18 years) with cranial oncology conditions undergoing surgery, regardless of additional treatments, and studies focused on pre- or postoperative nutritional assessment interventions (nutritional assessment tools, ONS, and nutritional counseling).

Exclusion criteria comprised book chapters, congress abstract, editorials, or studies with low methodological quality, and studies involving patients with metastases. This meticulous selection process was designed to ensure the scientific integrity and relevance of the sources included in the SR.

### 2.6. Risk of Bias and Methodological Quality Assessment

The risk of bias and methodological quality of included studies were independently assessed by two researchers (JCPV and GV) using the Joanna Briggs Institute (JBI) critical appraisal tools [41]. Recognized for their meticulousness in evaluating various research designs, these tools provided a structured framework to discern the reliability and applicability of each study. High-quality studies were identified based on a previous study [42], in which studies with a JBI score ≥ 70% were classified as high quality, those with a score between 70% and 50% as medium quality, and those with a score < 50% as low quality (Supplementary File S2).

### 2.7. Evidence Certainty Assessment

The certainty of evidence was evaluated using the Oxford Centre for Evidence-Based Medicine (OCEBM) framework [43], categorizing studies into five levels based on their design and quality. High-level studies, encompassing SRs of randomized controlled trials and superior individual trials, were conferred the first evidence tier. Conversely, research predominantly grounded in expert consensus or lacking empirical substantiation was categorized to the fifth level. Intermediate-level research, which includes but is not limited to less rigorous randomized controlled trials, cohort studies, and methodologies such as case series or case-control investigations, were allocated to the second, third, and fourth levels, respectively. Certain studies underwent a reassessment of their evidence level, either being elevated or diminished, influenced by parameters like methodological rigor, precision of findings, and the relevance of the results to the topic at hand [44].

### 2.8. Data Extraction and Synthesis

The following data from selected articles were extracted: authors, publication year, country, study design, population characteristics, nutritional interventions, and quality/bias evaluation. The extracted data were synthesized into a narrative summary, supplemented by figures and tables for clarity.

## 3. Results

### 3.1. Search Results

A total of 2884 records were identified through systematic searches across electronic databases, distributed as follows: PubMed (*n* = 1616), Embase (*n* = 359), CINAHL (*n* = 61), and Cochrane Library (*n* = 848). After removing 234 duplicates, 2650 unique titles were screened. Based on title relevance, 80 articles were selected for further evaluation through abstract review. During this phase, 25 articles were excluded as irrelevant. Subsequently, 55 full-text articles were assessed for eligibility. Of these, 45 studies were excluded for the following reasons: being systematic reviews (*n* = 8), lacking interventions related to nutritional assessment (*n* = 10), and being non-primary studies (*n* = 27). Additionally, 15 studies were identified through grey literature searches, of which 11 were excluded due to a lack of relevance to nutritional assessment, a focus on unrelated populations, or the inclusion of patients aged <18 years.

**Figure 1:**
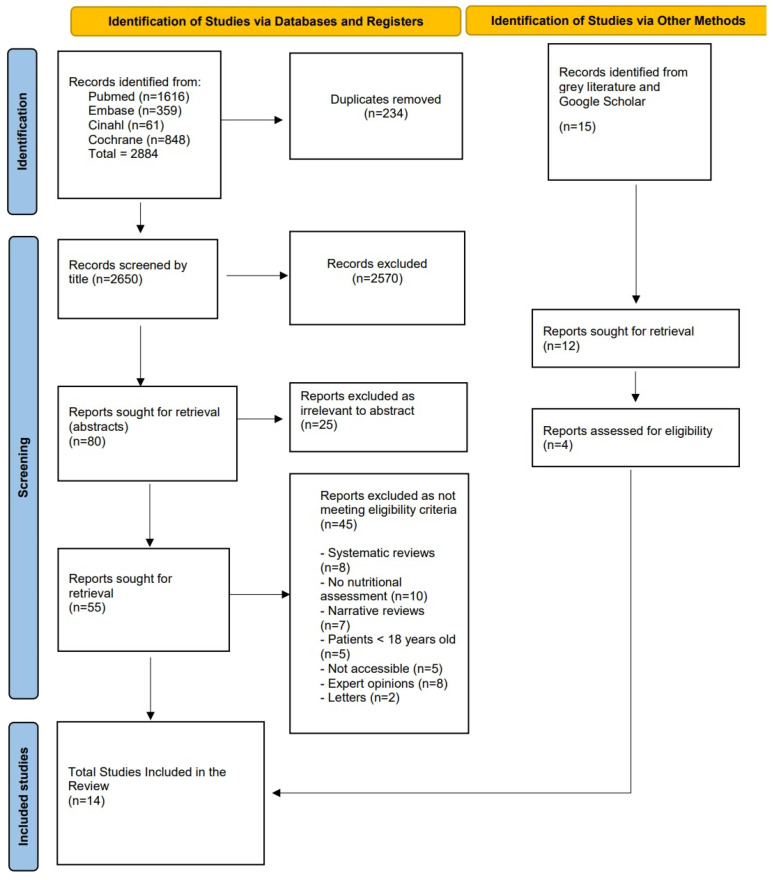
PRISMA flow diagram.

After the screening process, a total of 14 studies were included in this SR (Figure 1).

### 3.2. Characteristics of Studies, Population, and Interventions

This SR included a total of 14 studies, encompassing 11,224 patients (range: 1–9960). A significant proportion of these studies was conducted within the Chinese population (10,312 patients), followed by studies in South Korea (379 patients) and Italy (283 patients). In terms of study design, the included research comprised three randomized controlled trials (RCTs) [27,45,46], one quasi-experimental study [47], one prospective pilot study [48], seven cohort studies [26,29,30,49,50,51,52], one cross-sectional study [53], and one case report [54].

The methodological quality of the included studies was commendable, with a low risk of bias, as assessed using the JBI critical appraisal tools. A comprehensive overview of the key characteristics of the included studies is presented in Table 1.

### 3.3. Preoperative Nutritional Assessment

Seven studies [26,27,29,50,51,52,53] examined the importance of preoperative nutritional assessment in improving clinical outcomes in brain neoplasm patients undergoing neurosurgical procedures.

#### 3.3.1. Preoperative Nutritional Assessment Tools

The study by McCall et al. [53] utilized two diagnostic tools: the SGA and bioelectrical impedance analysis (BIA). The SGA, which incorporates parameters such as medical history, weight changes, dietary intake, gastrointestinal symptoms, functional status, and physical examination, classified 17.6% of the total sample of 109 patients as malnourished, with 94.7% moderately malnourished (SGA-B) and 5.3% severely malnourished (SGA-C). The BIA analysis revealed that patients with malignant tumors had a significantly higher lean body mass percentage (69.5% vs. 64.8%, *p* = 0.002) and lower fat mass percentage (30.4% vs. 35.2%, *p* = 0.033) compared to those with benign tumors. Additionally, malignancy was associated with greater preoperative weight loss (*p* = 0.038). Notably, 29.6% of patients were classified as overweight (BMI 25.0–29.9 kg/m^2^), and 32.4% were obese (BMI > 30.0 kg/m^2^), while six malnourished patients had a BMI < 18.5 kg/m^2^. Hu et al. [29] investigated the prognostic utility of the CONUT score, derived from albumin, total cholesterol, and lymphocyte count. An elevated CONUT score (≥4) was identified in 17.02% of the total sample of 94 patients and was associated with reduced overall survival (hazard ratio [HR]: 2.581; 95% confidence interval [CI]: 1.475–4.516, *p* = 0.001). Factors such as older age (≥60 years), absence of adjuvant treatments, and subtotal tumor resection were also predictors of poorer prognosis and shorter survival. These findings emphasize the prognostic value of the preoperative CONUT score and the importance of nutritional interventions for improving survival in GBM patients.

Several studies have evaluated preoperative albumin levels and the Prognostic Nutritional Index (PNI) as markers for nutritional status and survival outcomes. Han et al. [52] reported that preoperative albumin levels below 40 g/L were significantly associated with poor prognosis in GBM patients (95% CI: 0.938–0.995, *p* = 0.023), with a median overall survival of 14.0 months (95% CI: 11.7–14.3). Similarly, other studies [26,50,51] used the PNI, calculated from serum albumin and total lymphocyte count, to assess preoperative nutritional status. Huq et al. [26] demonstrated that patients with low albumin levels (<3.9 g/dL; 95% CI: 1.52–2.89, *p* < 0.001) and a low PNI (<43.38; 95% CI: 1.78–3.53, *p* < 0.001) experienced higher postoperative mortality, greater complications, and longer hospital stays compared to patients with high PNI values. Conversely, Zhou et al. [51] found that a PNI ≥ 44.4 was associated with improved prognosis and longer survival in GBM patients (HR: 0.479; 95% CI: 0.235–0.975, *p* = 0.042). However, Rigamonti et al. [50] questioned the reliability of the PNI as an independent predictor of survival, reporting no significant association between PNI and overall survival (HR: 0.90; 95% CI: 0.73–1.11, *p* = 0.32) (Table 2).

#### 3.3.2. Oral Nutritional Supplements

Liu et al. [27] evaluated the efficacy of preoperative administration of 400 mL of a maltodextrin-based nutritional supplement (200 kcal, 12.5% carbohydrates) two hours before surgery compared to standard fasting. The intervention group demonstrated improved postoperative glycemic homeostasis (5.6 ± 1.0 mmol/L vs. 6.3 ± 1.2 mmol/L, *p* = 0.001), greater handgrip strength (25.3 ± 7.1 kg vs. 19.9 ± 7.5 kg, *p* < 0.001), better pulmonary function (315.8 ± 91.5 L/min vs. 270.0 ± 102.7 L/min, *p* = 0.036), reduced complications, and faster recovery (*p* < 0.001 and *p* = 0.004, respectively). These findings underscore the benefits of preoperative nutritional support in optimizing patient condition before surgery and integrating oral nutritional supplements into standard preoperative protocols.

### 3.4. Postoperative Nutritional Assessment

Seven studies [30,45,46,47,48,49,54] emphasized the importance of continuous nutritional status monitoring to enhance recovery and prevent complications.

#### 3.4.1. Postoperative Nutritional Assessment Tools

Kim et al. [30] investigated the PNI both preoperatively (three weeks before surgery) and postoperatively (one week after surgery, before adjuvant therapy). The study revealed that low postoperative PNI values were associated with poorer survival outcomes (95% CI: 0.522–0.676; *p* = 0.018). Patients with a high postoperative PNI (≥50.2) demonstrated a longer median survival (19 months) compared to those with a low postoperative PNI (<50.2), whose median survival was 15 months (*p* < 0.001). These findings highlight the prognostic value of perioperative PNI changes in glioblastoma multiforme (GBM) patients. Xiao et al. [49] evaluated the association between perioperative serum albumin levels and clinical outcomes, such as complications and survival. Patients were categorized into four groups based on albumin variation (ΔA): ΔA < 5 g/L (normal, *n* = 2939, 30.4%), ΔA 5–9.9 g/L (mild, *n* = 4090, 42.3%), ΔA 10–14.9 g/L (moderate, *n* = 1959, 20.2%), and ΔA ≥ 15 g/L (severe, *n* = 681, 7%). Significant postoperative albumin reductions were associated with higher complication rates and poor prognosis, indicating the importance of postoperative nutritional monitoring and supplementation. Patients with mild reductions in albumin exhibited a 30-day mortality odds ratio of 1.84 (95% CI: 1.13–3.00; *p* = 0.014), with increased postoperative complications, such as infections and delayed recovery (odds ratio: 1.93; 95% CI: 1.17–3.18; *p* = 0.010).

#### 3.4.2. Oral Nutritional Supplements Combined with Nutritional Counseling

Five studies [45,46,47,48,54] explored the use of ONS combined with nutritional counseling by dietitians or nutritionists, demonstrating positive outcomes in improving rehabilitation and increasing protein and energy intake.

Dux et al. [47] introduced a postoperative protocol targeting dysphagia with protein-rich ONS (300 kcal/day, 200 mL at 1.5 kcal/mL), administered twice daily. This intervention significantly increased protein and caloric intake (58% vs. 93%, *p* < 0.001) while reducing malnutrition-related complications. Cho et al. [45] evaluated a protein-enriched ONS (72 kcal/day, 9 g of protein) administered twice daily for six weeks, combined with vitamins and minerals. The intervention improved muscle mass (measured by BIA) and rehabilitation outcomes compared to controls (*p* < 0.050), with no gastrointestinal side effects.

Wang et al. [46] applied an Enhanced Recovery After Surgery (ERAS) protocol involving preoperative and postoperative nutritional management. A polymeric supplement (200 kcal of maltodextrin and fructose in 400 mL) administered two hours before surgery significantly reduced hospital stay duration (4 vs. 7 days, *p* < 0.001) and accelerated recovery milestones, such as the time to first oral intake of liquids (4 vs. 8 h, *p* < 0.001) and solids (24 vs. 72 h, *p* < 0.001). Puri et al. [48] assessed postoperative supplementation with 8 mg/day of lycopene for six weeks, combined with chemoradiotherapy. Although progression-free survival was longer in the intervention group (40.83 weeks) compared to controls (26.74 weeks), the difference was not statistically significant (*p* = 0.089). Finally, Zuccoli et al. [54] documented a ketogenic diet combined with ONS and immunonutrition for 14 days post-surgery, showing benefits such as reduced inflammation, brain edema control, and absence of tumor recurrence during follow-up (Table 3).

### 3.5. Follow-Up

Follow-up periods varied among studies, reflecting the diverse impacts of nutritional interventions in neurosurgical patients. Cho et al. [45] reported a six-week follow-up with a 55% reduction in malnutrition in the intervention group. Wang et al. [46] followed patients for 30 days without re-hospitalizations or reinterventions. Long-term studies, such as Kim et al. [30] and Hu et al. [29], evaluated overall survival with follow-up periods of up to 19 months.

### 3.6. Involvement of Healthcare Professionals

Several studies [45,46,49] emphasized the role of nutritionists and dietitians in providing specialized nutritional counseling. Multidisciplinary teams, including neurosurgeons, anesthesiologists, and nurses, were involved in some studies [26,27,29]. However, some studies [50,52] did not specify dedicated nutritional professionals.

### 3.7. Summary of Evidence

This systematic review highlights the impact of nutritional assessment and interventions on clinical outcomes in neurosurgical patients. Regarding glucose homeostasis, one study [27] reported a significant reduction in postoperative fasting blood glucose levels following preoperative carbohydrate loading (5.6 ± 1.0 mmol/L vs. 6.3 ± 1.2 mmol/L, *p* = 0.001). In terms of inflammatory markers, Zuccoli et al. [54] reported a possible reduction in systemic inflammation, primarily inferred from the suppression of cerebral edema and discontinuation of corticosteroids. However, this observation is based on a single case report with low-level evidence, and no specific proinflammatory cytokines were directly measured. Functional recovery was mainly assessed through improvements in muscle mass, pulmonary function, and rehabilitation outcomes. Cho et al. [45] demonstrated that ONS administration led to increased muscle mass and enhanced rehabilitation (*p* < 0.050). Liu et al. [27] found improved handgrip strength (25.3 ± 7.1 kg vs. 19.9 ± 7.5 kg, *p* < 0.001) and better pulmonary function (315.8 ± 91.5 L/min vs. 270.0 ± 102.7 L/min, *p* = 0.036) in patients receiving preoperative carbohydrate load.

Nutritional counseling, often provided by dietitians and nutritionists, was integrated into several interventions but not consistently reported across studies. Dux et al. [47] highlighted its role in improving adherence to ONS protocols, leading to increased energy and protein intake (*p* < 0.001). Notably, ONS were considered an integral part of counseling in some cases, while in others, they were included in treatment protocols on demand.

Overall, these findings underscore the importance of structured nutritional interventions in improving metabolic regulation, reducing inflammation, and supporting postoperative recovery in neurosurgical patients. Future research should aim to standardize biochemical assessments and optimize nutritional strategies to enhance clinical outcomes.

## 4. Discussion

This systematic review highlights the critical role of pre- and postoperative nutritional assessment and interventions in improving outcomes for neurosurgical patients with brain neoplasms. The findings underscore that accurate preoperative nutritional evaluations, combined with targeted interventions, are essential to optimize clinical results, reduce postoperative complications, and accelerate recovery.

Several studies [45,53] demonstrated the effectiveness of tools like the SGA and BIA in identifying malnutrition risks before surgery. Consistent with these findings, Wobith et al. [55] emphasized that malnutrition is a significant prognostic factor in oncology, particularly in gastrointestinal cancers, advocating for standardized nutritional screening using tools like computed tomography (CT) and BIA. These findings align with the ESPEN guidelines, which emphasize the importance of early nutritional assessment and intervention. ESPEN recommends that nutritional evaluations be performed before surgery, using validated screening tools, combined with body composition assessments. The implementation of standardized nutritional screening protocols can facilitate the early detection of malnutrition, guiding targeted interventions to reduce surgical complications and enhance postoperative recovery [56,57].

The evidence supports integrating nutritional assessments into preoperative protocols. Malnutrition has been consistently linked to increased complications, delayed recovery, and reduced survival. De Pasquale et al. [58] demonstrated the benefits of combining assessment tools with nutritional prehabilitation strategies, which include personalized dietary counseling. Similarly, Hu et al. [29] confirmed the CONUT score as a reliable prognostic tool for short- and long-term outcomes, with higher scores linked to poorer survival rates. These findings suggest that integrating standardized nutritional evaluations can significantly enhance preoperative management.

Postoperative nutritional monitoring is equally critical. Two studies [26,51] showed correlations between reduced albumin levels and higher postoperative mortality and complications. Monitoring tools like the PNI provide valuable prognostic insights, as Kim et al. [30] found that a high postoperative PNI was associated with increased survival. Moreover, Xiao et al. [49] demonstrated that severe reductions in albumin levels were linked to higher complication rates, reinforcing the need for ongoing nutritional monitoring and interventions.

To ensure a systematic and individualized approach to nutritional care, patient management should follow the Nutrition Care Process (NCP) framework [59], which consists four essential steps: nutritional assessment, involving malnutrition screening using validated tools; nutritional diagnosis, aimed at identifying specific nutritional issues such as protein-energy malnutrition or cachexia; nutritional intervention, which consists of implementing targeted strategies like oral nutritional supplements, immunonutrition, or carbohydrate loading; and monitoring and evaluation, which assesses the patient’s response to intervention and allows for necessary adjustments to nutritional support. Integrating NCP-based strategies into perioperative protocols ensures personalized nutritional interventions, enhancing adherence and improving clinical outcomes [60]. The ESPEN guidelines advocate for a multidisciplinary approach, involving dietitians, neurosurgeons, and clinical nutritionists, to optimize perioperative nutritional management and support postoperative recovery in patients with brain neoplasms [56].

The integration of ONS with nutritional counseling has proven effective in enhancing rehabilitation and reducing malnutrition-related complications. Dux et al. [47] reported significant improvements in protein and caloric intake among patients using protein-rich ONS, while Wang et al. [46] demonstrated that ONS reduced hospital stay durations and accelerated recovery milestones, such as the resumption of oral intake. Furthermore, studies like Puri et al. [48] and Zuccoli et al. [54] highlighted the benefits of combining ONS with immunonutrition, which improved inflammation control, functional recovery, and quality of life.

Preoperative carbohydrate loading, as demonstrated in studies [61,62,63,64,65], also plays a pivotal role in reducing fasting-related stress, maintaining muscle mass, and enhancing recovery. Maltodextrin-based ONS, in particular, has shown benefits in improving postoperative glycemic control and reducing complications in neurosurgical patients and major surgery [36,66,67,68]. These findings suggest that tailored ONS interventions, combined with immunonutritional support, can significantly improve surgical outcomes.

The involvement of multidisciplinary teams, including neurosurgeons, dietitians, and nurses, is essential for implementing comprehensive nutritional strategies. Despite promising results, there remains a need to standardize protocols, particularly concerning the use of prognostic tools like the PNI and CONUT score. Further studies are required to refine these tools and validate their predictive capabilities across diverse patient populations.

Standardizing protocols and adopting a multidisciplinary approach will improve patient outcomes, reduce complications, and enhance quality of life. Future research should focus on optimizing nutritional strategies to address individual patient needs and establish evidence-based guidelines for clinical practice.

### 4.1. Multidisciplinary Approach and Future Directions

The integration of multidisciplinary teams, including surgeons, nutritionists, dietitians, and clinical nurse specialists (CNSs), is essential for effective nutritional management. CNSs, supported by dietitians, play a pivotal role in delivering interventions, monitoring nutritional needs, and providing education to patients and caregivers [69]. The advanced practice nurse (APN) framework further reinforces these efforts, emphasizing personalized care and mentorship to address barriers in nutritional management [70].

This review underscores the need to standardize nutritional protocols, particularly the application of tools such as the PNI and CONUT score, to ensure consistent and effective management. Tailored strategies aligned with a multidisciplinary approach are crucial for improving patient outcomes throughout the continuum of oncological care. Such integration not only optimizes recovery but also enhances the quality of life for patients undergoing neurosurgical procedures.

### 4.2. Study Limitations

This SR has several limitations. Many of the included studies had small sample sizes, potentially reducing statistical power and limiting the ability to detect significant differences between intervention groups. Additionally, most studies were retrospective, introducing selection bias and restricting causal inferences. The heterogeneity in study designs and nutritional protocols further complicates direct comparisons across studies.

A key limitation concerns the assessment of glucose homeostasis, which, although addressed in some studies, did not include comprehensive parameters such as insulin resistance (HOMA-IR) or insulin sensitivity indices, thereby limiting a complete understanding of metabolic regulation. Similarly, while some studies evaluated inflammatory responses, they lacked standardized assessments of proinflammatory cytokines (e.g., IL-6, TNF-α, IL-1β), making it difficult to accurately quantify the anti-inflammatory effects of nutritional interventions.

Another limitation is the scarcity of literature on the use of ONS and other nutrients in the preoperative phase, which may affect the generalizability of the findings. Additionally, nutritional counseling, although included in some studies, was not consistently reported in terms of methodology and adherence rates, emphasizing the need for standardized protocols to better assess its impact on clinical outcomes.

## 5. Conclusions

This SR underscores the critical role of pre- and postoperative nutritional assessment and interventions in managing neurosurgical patients. Malnourished patients, as identified through validated tools such as the CONUT score and PNI, consistently demonstrated higher complication rates and reduced survival. Preoperative nutritional interventions, including ONS and immunonutrition, have proven effective in optimizing nutritional status, modulating inflammatory responses, and reducing postoperative complications. Additionally, postoperative monitoring of nutritional indicators, such as serum albumin and PNI, further supported functional recovery and improved outcomes.

Overall, these findings emphasize the importance of adopting a multidisciplinary approach that integrates nutritional assessment as a core component of care for patients with brain neoplasms. However, heterogeneity in study designs and protocols highlights the urgent need for standardized guidelines to enhance the comparability and applicability of interventions across clinical settings. Future research should focus on refining nutritional strategies, validating the prognostic utility of specific indicators, and assessing their long-term impact on survival and quality of life. Targeted nutritional approaches could profoundly improve outcomes and advance clinical care in neurosurgical oncology.

## Figures and Tables

**Table 1:** Characteristics of the included studies.

Author	Country	Year	Study Design	Population (N)	Intervention Phase	Objective	Nutritional Intervention	Quality/Bias
Cho et al. [45]	Korea	2024	RCT	(*n* = 44) GP = 20 GC = 24	POST	Examined the effectiveness of ONS in improving functional outcomes and muscle strengthening in post-surgery patients.	ONS	High/+
Xiao et al. [49]	China	2024	Retrospective cohort study	(*n* = 9660)	POST	Investigated the association between pre- and postoperative albumin and outcomes in patients undergoing craniotomy for brain tumors.	Nutritional screening	High/+
Huq et al. [26]	USA	2021	Retrospective cohort study	(*n* = 242)	PRE	Examined the prognostic impact of preoperative nutritional status on postoperative outcomes in glioblastoma patients undergoing surgery.	Nutritional screening	High/+
Kim et al. [30]	Korea	2021	Retrospective cohort study	(*n* = 335)	POST	Evaluated the prognostic effect of postoperative PNI on outcomes in patients with GBM undergoing surgery.	Nutritional screening	High/+
Hu et al. [29]	China	2020	Retrospective cohort study	(*n* = 94)	PRE	Assessed the prognostic value of preoperative nutritional status in glioblastoma patients undergoing surgery.	Nutritional screening	High/+
Dux et al. [47]	Australia	2019	Quasi-experimental study	(*n* = 113) Pre-group = 55 Post-group = 58	POST	Implemented a nurse-led TFP to improve nutritional care post dysphagia assessment; transitioning from EN to ON.	ONS	High/+
Liu et al. [27]	China	2019	RCT	(*n* = 120) GI = 58 GC = 62	PRE	Assessed the effect of preoperative ONS versus fasting on postoperative outcomes in patients undergoing elective craniotomy.	ONS	High/+
Rigamonti et al. [50]	Italy	2019	Retrospective cohort study	(*n* = 282)	PRE	Evaluated the impact of PNI on OS in Italian glioblastoma patients.	Nutritional screening	High/+
Wang et al. [46]	China	2018	RCT	(*n* = 140) GC = 70 GI = 70	POST	Evaluated the safety and efficacy of a new ERAS protocol for patients undergoing elective craniotomies, and assessed postoperative hospital stay duration.	ONS	High/+
Zhou et al. [51]	China	2016	Retrospective cohort study	(*n* = 84)	PRE	Examined the significance of PNI as a prognostic factor in glioblastoma patients undergoing surgery.	Nutritional screening	High/+
Han et al. [52]	China	2015	Retrospective cohort study	(*n* = 214)	PRE	Verified the prognostic role of preoperative serum albumin levels and nutritional status in glioblastoma patients undergoing neurosurgery.	Nutritional screening	High/+
McCall et al. [53]	Canada	2014	Cross-sectional study	(*n* = 109)	PRE	Assessed the prevalence of malnutrition, nutritional status, and body composition in adult brain tumor patients awaiting resection.	Nutritional screening	High/+
Zuccoli et al. [54]	Italy	2010	Case report	(*n* = 1)	POST	Treatment of GBM with standard therapy and R-KD, supplemented by ONS and IM.	ONS	High/+
Puri et al. [48]	India	2010	Prospective pilot study	(*n* = 50) GI = 25 GC = 25	POST	Assessed the efficacy of lycopene as an adjunct treatment for high-grade gliomas alongside CT and RT post-surgery.	Carotenoids (lycopene)	High/+

Legend: RCT: randomized controlled trial; GBM: glioblastoma multiforme; ONS: oral nutritional supplements; IM: immunonutrition; R-KD: restricted ketogenic diet; TFP: transition food protocol; EN: enteral nutrition; ON: oral nutrition; OS: overall survival; PNI: Prognostic Nutritional Index; GI: intervention group; GC: control group; GP: protein group; CT: chemotherapy; RT: radiotherapy; ERAS: Enhanced Recovery After Surgery. Methodological quality and risk of bias were evaluated based on JBI criteria and Sguanci et al. [42]: High (+); Moderate (++); Low (+++).

**Table 2:** Preoperative nutritional assessment and treatment.

Author	Nutritional Intervention	Nutritional Management	Timing	Professionals Involved	Results	Adherence (%)	OCEBM Level
Huq et al. [26]	Nutritional screening	Use of PNI	Preoperative	Physicians; dietitians	Mean PNI = 47.4 ± 5.8; optimal PNI = 43.38 (22% of patients < 43.38) (HR, 2.51; 95% CI, 1.78–3.53; *p* < 0.001)	NR	4
Hu et al. [29]	Nutritional screening	CONUT score	Preoperative	Physicians; dietitians	OS = 1.475–4.516, *p* = 0.001	NR	4
Liu et al. [27]	ONS	ONS = 400 mL; 200 kcal; 12.5 g carbohydrates.	2 h before surgery in the GI	Neurosurgeons; nutritionist; nurses	Glucose homeostasis * (5.6 ± 1.0 mmol/L vs. 6.3 ± 1.2 mmol/L, *p* = 0.001); handgrip strength (25.3 ± 7.1 kg vs. 19.9 ± 7.5 kg, *p* < 0.001); pulmonary function (315.8 ± 91.5 L/min vs. 270.0 ± 102.7 L/min, *p* = 0.036); reduced hospital stay (−3 days, *p* < 0.001 and *p* = 0.004)	100%	2
Rigamonti et al. [50]	Nutritional screening	Use of PNI	NR	Neurosurgeons; oncologists	Median PNI = 46.9 (CI 26.2–72.5); PNI/OS association *p* = 0.32	NR	3
Zhou et al. [51]	Nutritional screening	Use of PNI	NR	Oncologists; neurosurgeons; nutritionists	PNI ≥ 44.4 (HR: 0.479, 95% CI: 0.235–0.975, *p* = 0.042)	NR	4
Han et al. [52]	Nutritional screening	Albumin, prealbumin, lymphocyte levels	Preoperative	Oncologists; neurosurgeons	Median OS = 14.0 (95% CI 11.7–14.3) months; multivariate HR for OS = 0.966, 95% CI 0.938–0.995, *p* = 0.023; preoperative albumin = 37.4 ± 5.6 g/L, *p* < 0.001	100%	4
McCall et al. [53]	Nutritional screening	Assessment tools: SGA-A; SGA-B; SGA-C; BIA	Preoperative	Neurosurgeon; dietitian; nurses **	Malnutrition = 17.6%; malignant tumors = 30.4% (fat mass) vs. 35.2% (lean mass), *p* = 0.033	NR	4

Legend: PNI: Prognostic Nutritional Index (10 × albumin (g/dL) + 0.005 × total lymphocyte count); SGA: Subjective Global Assessment; SGA-A: normally nourished; SGA-B: slightly or moderately malnourished; SGA-C: severely malnourished; BIA: bioelectrical impedance analysis; CONUT: preoperative checking nutritional status; GI: intervention group; OS: overall survival; ONS: oral nutritional supplements; MG: fat mass; MM: lean mass; NR: not reported; *p*: *p* Value; *: blood glucose levels (mmol/L); insulin levels (μunits/mL); nurses **: Responsible for evaluation and symptom identification through screening tools; evidence level based on the Oxford Centre for Evidence-Based Medicine (OCEBM) framework [43].

**Table 3:** Postoperative nutritional assessment and treatment.

Author	Nutritional Intervention	Nutritional Management	Timing & Follow-Up	Professionals Involved	Results	Adherence (%)	OCEBM Level
Cho et al. [45]	ONS	ONS = 100 mL; 72 kcal; 36 g protein	Timing: Twice daily for 6 weeks post-surgery; Follow-up: Present	Nutritionist	Association between ONS and improved muscle mass and rehabilitation outcomes compared to GC (*p* < 0.050)	90%	2
Xiao et al. [49]	Nutritional screening	Albumin levels evaluated in 4 groups	Timing: Within 14 days post-surgery; Follow-up: Present for 30 days	Neurosurgeons; anesthetists; nutritionists; nurses	Association between albumin reduction and increased 30-day mortality (odds ratio 1.84; 95% CI, 1.13–3.00; *p* = 0.014)	98.2%	3
Kim et al. [30]	Nutritional screening	PNI evaluation	Timing: 1 week post-surgery; Follow-up: Present	Neurosurgeons; clinical nutritionist	Low PNI associated with worse OS (95% CI: 0.522–0.676; *p* = 0.018)	NR	4
Dux et al. [47]	ONS	ONS = 200 mL; 300 kcal; protein NR	Timing: Two servings/day; Follow-up: Present	Nurses; dietitians; physicians; speech therapists	Improved protein and energy intake in the post-implementation group (*p* < 0.001)	79%	3
Wang et al. [46]	ONS	ONS = 400 mL; 300 kcal; carbohydrates NR	Timing: 8 h post-surgery; Follow-up: Present	Neurosurgeons; anesthetists; nurses; physiotherapists; nutritionists	Reduced hospital stay (approx. 4 days vs. 7 days; *p* < 0.001); earlier oral polymeric nutritional drink intake (median 8 h vs. 11 h, *p* < 0.001)	80%	2
Zuccoli et al. [54]	ONS	ONS = NR ml; 600 kcal; 20 g protein IM = 10 g omega-3; kcal NR	Timing: 2 months; Follow-up: Present	Neurologist; nutritionist	Decreased inflammatory activity; BMI reduction; no tumor tissue detected; reduced glucose levels	100%	5
Puri et al. [48]	Oral lycopene supplementation	Plasma lycopene levels assessed pre- and post-radiotherapy using HPLC	Timing: Oral lycopene 8 mg/day for 6 weeks with radiotherapy. Plasma lycopene measured before and after treatment	Oncologist; nutritionist	Time to progression in IG vs. CG: 40.83 weeks vs. 26.74 weeks (*p* = 0.089); not significant, despite a longer time to progression in the IG	100%	4

Legend: ONS: oral nutritional supplement; PNI: Prognostic Nutritional Index; OS: overall survival; IM: immunonutrition; CG: control group; IG: intervention group; NR: not reported; BMI: Body Mass Index; CI: confidence interval. Evidence level based on the Oxford Centre for Evidence-Based Medicine (OCEBM) framework [43].

## Data Availability

The data from this systematic review are fully made available in the manuscript text and Supplementary Files.

## Supplementary Materials

The following supporting information can be downloaded at: https://www.mdpi.com/article/10.3390/cancers17050764/s1, Supplementary File S1 (Table S1 Search strategy); Supplementary File S2 (Tables S2–S6 JBI Critical Appraisal).
