# Supplementary material for: Nutritional Assessment and Management of Patients with Brain Neoplasms Undergoing Neurosurgery: A Systematic Review
Source: Cancers (Basel). 2025 Feb 24;17(5):764. doi: 10.3390/cancers17050764 (PMC11898651; doi:10.3390/cancers17050764)
Supplement: Supplementary file 1 [file cancers-17-00764-s001.zip › File supplementary S1.pdf]

File supplementary S1

Table S1. *Search Strategy*

ID	PUBMED	Results
#1	("Brain Neoplasms"[MeSH Terms] OR "brain neoplasms*"[All Fields]) OR (Glioblastoma [MeSH Terms] OR glioblastoma* [All Fields]) OR (Astrocytoma [MeSH Terms] OR astrocytoma* [All Fields]) OR (Meningioma [MeSH Terms] OR meningioma* [All Fields]) OR (Glioma [MeSH Terms] OR glioma* [All Fields]) OR "brain tumor*"[All Fields] OR "encephalic tumor*"[All Fields] OR "brain surgery*"[All Fields] OR (Neurosurgery [MeSH Terms] OR neurosurgery*[All Fields])	
#2	("Nutritional Status"[MeSH Terms] OR "nutritional status*"[All Fields]) OR ("Body Composition"[MeSH Terms] OR "body composition*"[All Fields]) OR ("Nutrition Assessment"[MeSH Terms] OR "nutrition assessment*"[All Fields]) OR ("Body Mass Index"[MeSH Terms] OR "body mass index*"[All Fields]) OR (Diet [MeSH Terms] OR diet* [All Fields]) OR ("diet, food, and nutrition"[MeSH Terms] OR "diet food and nutrition*"[All Fields]) OR nutrition*[All Fields]	
#3	("Patient Care"[MeSH Terms] OR "patient care*"[All Fields]) OR ("Nutritional Support"[MeSH Terms] OR "nutritional support*"[All Fields]) OR ("Nutrition Therapy"[MeSH Terms] OR "nutrition therapy*"[All Fields]) OR ("Perioperative Care"[MeSH Terms] OR "perioperative care*"[All Fields]) OR prehabilitation*[All Fields] OR "nutritional prehabilitation*"[All Fields] OR "pre habilitation*"[All Fields] OR ("Preoperative Care"[MeSH Terms] OR "preoperative care*"[All Fields]) OR "pre operative*"[All Fields]	
**#4**	**#1 AND #2 AND #3**<br>((("Brain Neoplasms"[MeSH Terms] OR "brain neoplasms*"[All Fields]) OR ("glioblastoma"[MeSH Terms] OR "glioblastoma*"[All Fields]) OR ("astrocytoma"[MeSH Terms] OR "astrocytoma*"[All Fields]) OR ("meningioma"[MeSH Terms] OR "meningioma*"[All Fields]) OR ("glioma"[MeSH Terms] OR "glioma*"[All Fields]) OR "brain tumor*"[All Fields] OR "encephalic tumor*"[All Fields] OR "brain surgery*"[All Fields] OR ("neurosurgical procedures"[MeSH Terms] OR "neurosurgery"[MeSH Terms] OR "neurosurgery*"[All Fields])) AND (("Nutritional Status"[MeSH Terms] OR "nutritional status*"[All Fields]) OR ("Body Composition"[MeSH Terms] OR "body composition*"[All Fields]) OR ("Nutrition Assessment"[MeSH Terms] OR "nutrition assessment*"[All Fields]) OR ("Body Mass Index"[MeSH Terms] OR "body mass index*"[All Fields]) OR ("diet"[MeSH Terms] OR "diet*"[All Fields]) OR ("diet, food, and nutrition"[MeSH Terms] OR "diet food and nutrition*"[All Fields]) OR "nutrition*"[All Fields]) AND (("Patient Care"[MeSH Terms] OR "patient care*"[All Fields]) OR ("Nutritional Support"[MeSH Terms] OR "nutritional support*"[All Fields]) OR ("Nutrition Therapy"[MeSH Terms] OR "nutrition therapy*"[All Fields]) OR ("Perioperative Care"[MeSH Terms] OR "perioperative care*"[All Fields]) OR "prehabilitation*"[All Fields] OR "nutritional prehabilitation*"[All Fields] OR "pre habilitation*"[All Fields] OR ("Preoperative Care"[MeSH Terms] OR "preoperative care*"[All Fields]) OR "pre operative*"[All Fields]))	**1.616**
	**COCHRANE**	**Results**
#1	Brain Neoplasms* OR Glioblastoma* OR Astrocytoma* OR Meningioma* OR Glioma* OR Brain Tumor* OR Brain Surgery* OR Neurosurgery*	

#2	Nutritional Status* OR Body Composition* OR Nutrition Assessment* OR Body Mass Index* OR Diet* OR Diet Food and Nutrition* OR Nutrition*	
#3	Patient Care* OR Nutritional Support* OR Nutrition Therapy* OR Perioperative Care* OR Prehabilitation* OR Nutritional Prehabilitation* OR Pre Habilitation* OR Preoperative Care* OR Pre Operative*	
**#4**	**#1 AND #2 AND #3**<br>**(Brain Neoplasms* OR Glioblastoma* OR Astrocytoma* OR Meningioma* OR Glioma* OR Brain Tumor* OR Brain Surgery* OR Neurosurgery*) AND (Nutritional Status* OR Body Composition* OR Nutrition Assessment* OR Body Mass Index* OR Diet* OR Diet Food and Nutrition* OR Nutrition*) AND (Patient Care* OR Nutritional Support* OR Nutrition Therapy* OR Perioperative Care* OR Prehabilitation* OR Nutritional Prehabilitation* OR Pre Habilitation* OR Preoperative Care* OR Pre Operative*)**	
		**848**
**ID**	**Cumulative Index Of Nursing And Allied Health Literature (CINAHL)**	**Results**
#1	"brain neoplasms*" OR glioblastoma* OR astrocytoma* OR meningioma* OR glioma* OR "brain tumor*" OR "encephalic tumor*" OR "brain surgery*" OR neurosurgery*	
#2	"nutritional status*" OR "body composition*" OR "nutrition assessment*" OR "body mass index*" OR diet* OR "diet food and nutrition*" OR nutrition*	
#3	"patient care*" OR "nutritional support*" OR "nutrition therapy*" OR "perioperative care*" OR prehabilitation* OR "nutritional prehabilitation*" OR "pre habilitation*" OR "preoperative care*" OR "pre operative*"	
**#4**	**#1 AND #2 AND #3**<br>**("brain neoplasms*" OR glioblastoma* OR astrocytoma* OR meningioma* OR glioma* OR "brain tumor*" OR "encephalic tumor*" OR "brain surgery*" OR neurosurgery*) AND ("nutritional status*" OR "body composition*" OR "nutrition assessment*" OR "body mass index*" OR diet* OR "diet food and nutrition*" OR nutrition*) AND ("patient care*" OR "nutritional support*" OR "nutrition therapy*" OR "perioperative care*" OR prehabilitation* OR "nutritional prehabilitation*" OR "pre habilitation*" OR "preoperative care*" OR "pre operative*")**	**61**
**ID**	**EMBASE**	
#1	'brain tumor' OR 'brain tumor*' OR glioblastoma OR glioblastoma* OR astrocytoma OR astrocytoma* OR meningioma OR meningioma* OR glioma OR glioma* OR 'brain surgery' OR 'brain surgery*'	
#2	'nutritional status' OR 'nutritional status*' OR 'body composition' OR 'body composition*' OR 'nutritional assessment' OR 'nutritional assessment*' OR 'body mass' OR 'body mass*' OR diet OR diet* OR nutrition OR nutrition*	
#3	'patient care' OR 'patient care*' OR 'nutritional support' OR 'nutritional support*' OR 'diet therapy' OR 'diet therapy*' OR 'preoperative care' OR 'preoperative care*' OR 'preoperative exercise' OR	

	'preoperative exercise*'	
**#4**	**#1 AND #2 AND #3**<br><br>**('brain tumor' OR 'brain tumor*' OR glioblastoma OR glioblastoma* OR astrocytoma OR astrocytoma* OR meningioma OR meningioma* OR glioma OR glioma* OR 'brain surgery' OR 'brain surgery*') AND ('nutritional status' OR 'nutritional status*' OR 'body composition' OR 'body composition*' OR 'nutritional assessment' OR 'nutritional assessment*' OR 'body mass' OR 'body mass*' OR diet OR diet* OR nutrition OR nutrition*) AND ('patient care' OR 'patient care*' OR 'nutritional support' OR 'nutritional support*' OR 'diet therapy' OR 'diet therapy*' OR 'preoperative care' OR 'preoperative care*' OR 'preoperative exercise' OR 'preoperative exercise*')**	**359**
**TOTAL ARTICLES FOUND**		**2,884**
