# Supplementary material for: Nutritional Assessment and Management of Patients with Brain Neoplasms Undergoing Neurosurgery: A Systematic Review
Source: Cancers (Basel). 2025 Feb 24;17(5):764. doi: 10.3390/cancers17050764 (PMC11898651; doi:10.3390/cancers17050764)
Supplement: Supplementary file 1 [file cancers-17-00764-s001.zip › File supplementary S2.pdf]

File supplementary S2

Table S2: *JBI Critical appraisal tool for RCT*

STUDY	ITEM 1	ITEM 2	ITEM 3	ITEM 4	ITEM 5	ITEM 6	ITEM 7	ITEM 8	ITEM 9	ITEM 10	ITEM 11	ITEM 12	ITEM 13	INCLUDE	SCORE
Cho et al. Corea 2024	Y	Y	Y	Y	Y	Y	Y	Y	Y	Y	Y	Y	Y	Y	100%
Liu et al. Cina 2019	Y	Y	Y	N	N	N	Y	Y	Y	Y	Y	Y	Y	Y	77%
Wang et al. Cina 2018	Y	Y	Y	N	N	N	Y	Y	Y	Y	Y	Y	Y	Y	77%

Table S2. JBI Critical appraisal tool for Randomized Controlled Trials

Legend: Y=Yes; N=No; U=Unclear; NA=Not Applicable / Items from *JBI Critical appraisal tool for Randomized Controlled Trials: 1. Was true randomization used for assignment of participants to treatment groups? 2.Was allocation to treatment groups concealed? 3. Were treatment groups similar at the baseline? 4. Were participants blind to treatment assignment?5. Were those delivering treatment blind to treatment assignment? ;6.Were outcomes assessors blind to treatment assignment?;7.Were treatment groups treated identically other than the intervention of interest?;8.Was follow up complete and if not, were differences between groups in terms of their follow up adequately described and analyzed?;9.Were participants analyzed in the groups to which they were randomized?;10.Were outcomes measured in the same way for treatment groups?;11.Were outcomes measured in a reliable way?;12.Was appropriate statistical analysis used?;13.Was the trial design appropriate, and any deviations from the standard RCT design (individual randomization, parallel groups) accounted for in the conduct and analysis of the trial?*

Table S3: *JBI Critical appraisal tool for quasi-experimental studies*

STUDY	ITEM 1	ITEM 2	ITEM 3	ITEM 4	ITEM 5	ITEM 6	ITEM 7	ITEM 8	ITEM 9	INCLUDE	SCORE
Dux et al.	Y	Y	Y	N	Y	NA	Y	Y	Y	Y	77.7%
Puri et al.	Y	Y	Y	Y	Y	N	Y	Y	Y	Y	89%

Table S3. JBI Critical appraisal tool for quasi-experimental studies

Legend: Y=Yes; N=No; U=Unclear; NA=Not Applicable / Items from *JBI Critical appraisal tool for quasi-experimental studies:1=Is it clear in the study what is the 'cause' and what is the 'effect' (i.e. there is no confusion about which variable comes first)?; 2=Were the participants included in any comparisons similar?; 3=Were the participants included in any comparisons receiving similar treatment/care, other than the exposure or intervention of interest?; 4=Was there a control group?; 5=Were there multiple measurements of the outcome both pre and post the intervention/exposure?; 6=Was follow up complete and if not, were differences between groups in terms of their follow up adequately described and analyzed?; 7=Were the outcomes of participants included in any comparisons measured in the same way?;8=Were outcomes measured in a reliable way?; 9=Was appropriate statistical analysis used?*

Table S4: *Critical appraisal tool of analytical cross sectional studies*

STUDY	ITEM 1	ITEM 2	ITEM 3	ITEM 4	ITEM 5	ITEM 6	ITEM 7	ITEM 8	INCLUDE	SCORE
McCall et al.	Y	Y	Y	Y	U	U	Y	Y	Y	75%

Table S4 JBI. Critical appraisal tool of analytical cross sectional studies

Legend: Y=Yes; N=No; U=Unclear; NA=Not Applicable / Items from Critical appraisal of analytical cross sectional studies: 1= Were the criteria for inclusion in the sample clearly defined? 2=Were the study subjects and the setting described in detail?, 3=Was the exposure measured in a valid and reliable way?, 4=Were objective, standard criteria used for measurement of the condition?, 5=Were confounding factors identified?, 6=Were strategies to deal with confounding factors stated?, 7=Were the outcomes measured in a valid and reliable way?, 8=Was appropriate statistical analysis used?

Table S5: *Critical appraisal of cohort studies included*

STUDY	ITEM 1	ITEM 2	ITEM 3	ITEM 4	ITEM 5	ITEM 6	ITEM 7	ITEM 8	ITEM 9	ITEM 10	ITEM 11	INCLUDE	SCORE
Xiao et al.	Y	Y	Y	Y	Y	Y	Y	Y	Y	U	Y	Y	90,9%
Huq et al.	Y	Y	Y	Y	Y	Y	Y	Y	NA	NA	Y	Y	81,8%
Kim et al.	NA	NA	Y	Y	Y	Y	Y	Y	Y	Y	Y	Y	81.8%
Hu et al.	Y	Y	Y	Y	Y	Y	Y	Y	NA	NA	Y	Y	81.8%
Rigamonti et al.	Y	Y	Y	Y	Y	Y	Y	Y	Y	U	Y	Y	90,9%
Zhou et al.	Y	Y	Y	Y	Y	Y	Y	Y	Y	NA	Y	Y	90,9%
Han et al.	Y	Y	Y	Y	Y	Y	Y	Y	Y	NA	Y	Y	90,9%

Table S5. Critical appraisal of cohort studies included

Legend: Y=Yes; N=No; U=Unclear; NA=Not Applicable; / Items from JBI Critical appraisal checklist for Cohort Study: 1= Were the two groups similar and recruited from the same population? 2= Were the exposures measured similarly to assign people to both exposed and unexposed groups? 3= Was the exposure measured in a valid and reliable way? 4= Were confounding factors identified? 5= Were strategies to deal with confounding factors stated? 6= Were the groups/participants free of the outcome at the start of the study (or at the moment of exposure)? 7= Were the outcomes measured in a valid and reliable way? 8= Was the follow-up time reported and sufficient to be long enough for outcomes to occur? 9= Was follow-up complete, and if not, were the reasons to loss to follow up described and explored? 10= Were strategies to address incomplete follow-up utilized? 11= Was appropriate statistical analysis used?

Table S6: *Critical appraisal CHECKLIST of case reports*

STUDY	ITEM 1	ITEM 2	ITEM 3	ITEM 4	ITEM 5	ITEM 6	ITEM 7	ITEM 8	INCLUDE	SCORE
Zuccoli et al.	Y	Y	Y	Y	Y	Y	Y	Y	Y	100%

Table S6. Critical appraisal CHECKLIST of case reports

Legend: Y=Yes; N=No; U=Unclear; NA=Not Applicable; / Items from JBI Critical appraisal checklist for Qualitative studies: 1. Were patients's demographic characteristics clearly described?; 2. Was the patient's history clearly described and presented as a timeline?; 3. Was the current clinical condition of the patients on presentation clearly described?; 4. Were diagnostic tests or assenssment methods and the results clearly described?; 5. Was the intervention(s) or treatment procedure(s) clearly described?; 6. Was the post-intervention clinical condition clearly described?; 7. Were adverse events (harms) or unanticipated events identified and described?; 8. Does the case report provide takeway lessons?
